# Using RNA-Seq Analysis to Select Key Genes Related to Seed Dormancy in ALS-Inhibiting Resistant *Descurainia sophia* with Pro-197-Thr Mutation

**DOI:** 10.3390/plants13162305

**Published:** 2024-08-19

**Authors:** Xian Xu, Bochui Zhao, Beibei Shen, Zhizun Qi, Jianping Wang, Haiyan Cui, Binghua Li, Silong Chen, Guiqi Wang, Xiaomin Liu

**Affiliations:** 1 Key Laboratory of Crop Cultivation Physiology and Green Production of Hebei Province, Institute of Cereal and Oil Crops, Hebei Academy of Agriculture and Forestry Sciences, Shijiazhuang 050035, China; xuxian19790801@163.com (X.X.); zhaobochui@163.com (B.Z.); s15100158641@126.com (B.S.); qizz783@163.com (Z.Q.); wang_jp1@163.com (J.W.); cuihy-1@163.com (H.C.); binghua-li@163.com (B.L.); 2 College of Food Science and Biology, Hebei University of Science and Technology, Shijiazhuang 050018, China

**Keywords:** resistant flixweed, Pro-197-Thr, dormancy mechanism, *CYP707A2*

## Abstract

Flixweed (*Descurainia sophia*) is a weed that seriously affects wheat fields in China. Over the past 20 years, it has evolved resistance to the herbicide tribenuron-methyl. In the present study, a resistant *D. sophia* population with a Pro-197-Thr mutation of acetolactate synthetase (ALS) was found to have a resistance index of 457.37 for tribenuron-methyl. Under the same growth conditions, the seeds of resistant (R) and susceptible (S) populations exhibited similar vitality but the germination rates of R seeds were higher than those of S seeds. This result demonstrated that seed dormancy periods were shorter in the R seeds. RNA-Seq transcriptome analysis was then used to choose candidate genes that could regulate seed dormancy pathways in the R population. A total of 504,976,046 clean reads were selected from nine RNA-Seq libraries and assembled into 79,729 unigenes. Among these, 33,476 unigenes were assigned to 51 GO subgroups, and 26,117 unigenes were assigned to 20 KEGG secondary metabolic pathways. Next, 2473 differentially expressed genes (DEGs) were divided into three groups, as follows: G-24 h (germinating seeds) vs. D (dormant seeds); G-48 h (germinated seeds) vs. D; and G-48 h vs. G-24 h. From these 2473 DEGs, 8 were selected as candidate dormancy unigenes for the R population if their expression levels continuously decreased during the seed germination progress and their functional annotations were related to plant seed dormancy. One candidate unigene was annotated as *CYP707A2*; two unigenes were annotated as the transcription factors *TGA4* and *TGA2*; one unigene was annotated as the cystathionine beta-synthase gene; and four unigenes could not be annotated as any gene listed in the six public databases. However, qRT-PCR-validated results showed that, during the germination of R seeds, the expression of the three candidate unigenes first decreased and then increased, indicating that they may have other growth-regulating functions in R populations. In brief, the dormancy function of the eight candidate dormancy unigenes needs to be further studied.

## 1. Introduction

Weeds are a major cause of reduced crop production. Crop yields from fields affected by weeds are typically 30 percent lower than yields from unaffected fields, and some fields affected by weeds fail to yield any grain at all [1]. The prevalence of weeds in crop fields is related to their seed dormancy characteristic [2]. Seed dormancy is one of the mechanisms by which weeds adapt themselves to various environmental conditions [3]. Dormancy is formative during the process in which seeds become ripe, and it determines whether germination occurs periodically or is delayed so that the species does not face extinction as a result of simultaneous germination under hostile natural conditions to which the plants are unable to adapt [4,5].

Dormancy in plant seeds is regulated in vivo by hormones; among these, abscisic acid (ABA) is a positive regulator of seed dormancy induction and maintenance [6]. Changes in the expression of genes that participate in ABA biosynthesis, metabolism, and signaling pathways have been shown to inhibit and delay seed germination [7,8,9]. Some dormancy genes have been discovered in different plants. For instance, *DESPIERTO* and *ATHB20*, both of which are involved in ABA sensitivity, were found to be overexpressed in after-ripened seeds of *Arabidopsis thaliana*, and this finding was proven to be related to seed dormancy regulation [10]. The DELAY OF GERMINATION1 gene (*DOG1*) is another seed dormancy gene that has been identified in *A. thaliana* [11]. The authors of [12] found that *DOG1* expression increased in mature *A. thaliana* seeds. In addition, the *DOG1* homologous genes *TaDOG1L1* and *HvDOG1L1* have been shown to control seed dormancy in both wheat (*Triticum aestivum*) and barley (*Hordeum vulgare*) [13]. 

Flixweed (*Descurainia sophia*) is a dicotyledonous weed of the Cruciferae family, which is widely distributed in wheat fields in northern China [14]. *D. sophia* may be considered a particularly troublesome weed because it competes with wheat for water, sunshine, and nutrition [15]. *D. sophia* seeds exhibit a strong dormancy characteristic. When freshly harvested, they do not germinate. Instead, there is an after-ripening period that lasts for about 6 months. *D. sophia* seeds then break their dormancy and begin to germinate, with a seed germination rate of about 90% [16]. Over the past 30 years, tribenuron-methyl, an acetolactate synthetase (ALS) inhibitor, has been the most important herbicide used for the control of *D. sophia* in China [17]. Today, most of the *D. sophia* populations in Chinese wheat fields have evolved high levels of resistance to tribenuron-methyl. The results of three recent studies [18,19,20] showed ALS mutations at 197, 376, and 574 positions, respectively. Other research results have shown that the mechanisms of seed dormancy are very complex, with different species having different seed dormancy genes [21,22]. To date, however, the mechanisms of seed dormancy in resistant populations of *D. sophia* have not been identified by researchers.

Whole-transcriptome sequencing (RNA-Seq) is a powerful form of technology, used today to discover putative genes that take part in certain biological responses [23]. A number of non-target-site resistance genes have been found using this technology. Four *CytP450* genes, *CYP94A1*, *CYP94A2*, *CYP71A4*, and *CYP734A6* were discovered in shortawn foxtail (*Alopecurus aequalis*) and shown to be related to metabolic resistance by the authors of [24]. In another study, *CYP96A146* was found to be overexpressed in a resistant *D. sophia* population after spraying with tribenuron-methyl [25]. In addition, unigenes controlling dormancy in buds have been discovered in *Lilium pumilum* [26], Japanese pear (*Pyrus pyrifolia*) [27], and Chinese white pear (*Pyrus pyrifolia*) [28]. 

In the present study, differences in the seed germination rates of susceptible and highly resistant *D. sophia* populations with ALS mutation were studied, and RNA-Seq transcriptome analysis and qRT-PCR technology were used to determine the putative genes involved in the dormancy pathways of seeds in the resistant *D. sophia* population. 

## 2. Results

### 2.1. Whole-Plant Bioassay of Suspected Resistant D. sophia Population

**Figure 1:**
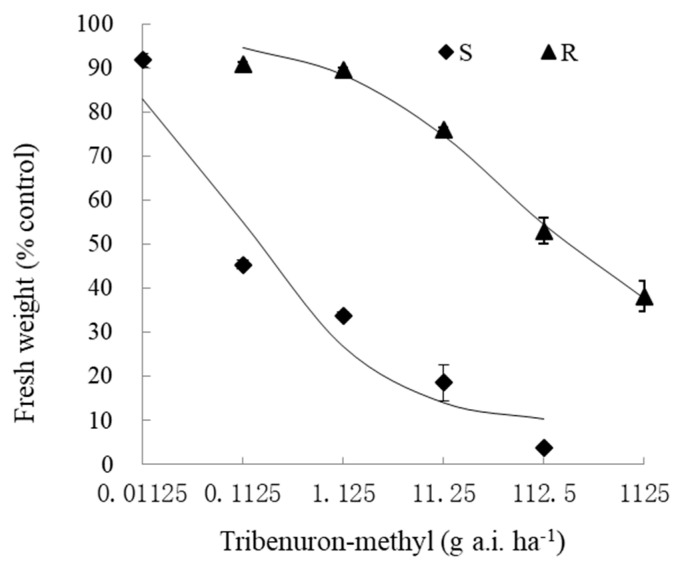
Whole-plant dose–response curves for flixweed (*Descurainia sophia*) populations that were either S (susceptible) or R (resistant) to tribenuron-methyl. Each value represents a mean of fresh weight (%control) ± standard error.

The results of the whole-plant bioassay showed that the suspected R population had evolved a high resistance to tribenuron-methyl. The GR_50_ values for the S and R populations were found to be 0.12 and 54.88 g a.i. ha^−1^, respectively, and the resistance index of R was calculated to be 457.34 (Table 1 and Figure 1). 

### 2.2. Analysis of ALS Gene Sequences in R and S Populations

**Figure 2:**
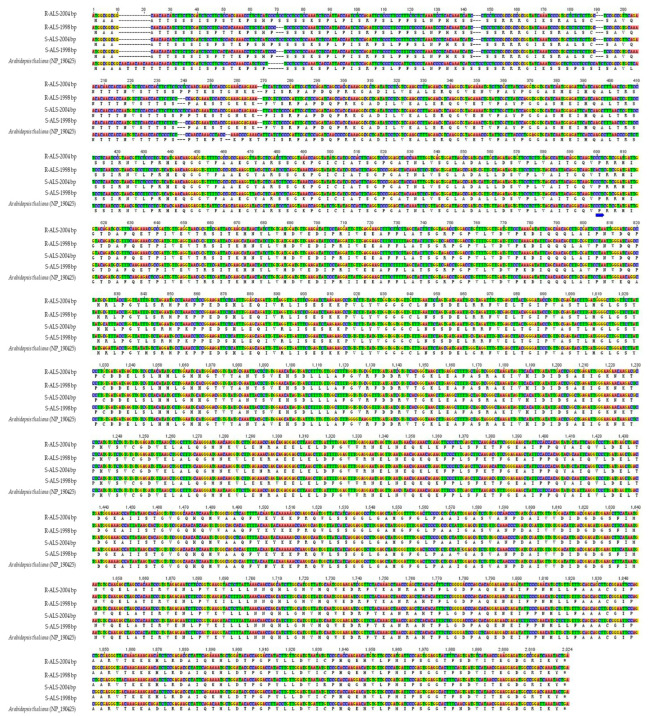
Homologous alignment of ALS DNA and amino acid sequences of R, S, and *Arabidopsis thaliana*. The codon of the 197-Pro was CCT in *A. thaliana.*

Two *ALS* genes were cloned from the R and S populations, with lengths of 1998 bp and 2004 bp, respectively, of their gene coding regions (Figure 2). Analysis of the DNA and amino acid sequences revealed a mutation, Pro-197-Thr (related to the ALS amino acid site of *Arabidopsis thaliana*), in one of the *ALS* genes (1998 bp) in the R population. This mutation has previously been associated with the evolution of resistance to ALS-inhibiting herbicides in weeds. In addition, we detected no ALS mutation related to such resistance in the coding regions of the two *ALS* genes in the S population (Figure 2). Taken together with the results of the whole-plant bioassay described above, this finding confirmed that the R population was a highly resistant biotype.

### 2.3. Differences in Seed Germination between R and S Populations

Freshly ripened seeds of the R and S populations exhibited no germination when treated with water. However, when R and S seeds were forced to break dormancy by being immersed in 0.1%GA3, their germination rates were 88% and 86%, respectively. This result demonstrated that the freshly ripened R and S seeds exhibited similar levels of vitality (Table 2). Contrarily, when the seeds of R and S populations were allowed to break dormancy and began to germinate naturally, their germination rates were found to differ significantly, 62% and 6%, respectively (Table 2). These results demonstrate that R and S populations were characterized by different seed dormancy periods.

### 2.4. Mining Candidate Dormant Genes from R Population Using RNA-Seq 

#### 2.4.1. Illumina Sequencing and De Novo Assembly

A total of 508,375,818 raw reads were generated from nine sample libraries (D, G-24 h, and G-48 h, each with three biological replicates) using Illumina sequencing technology. Next, 504,976,046 clean reads were selected from the raw reads. The number of clean reads ranged from 49,610,034 to 65,670,676. After quality control, the clean reads were assembled into 79,729 unigenes and 164,087 transcripts. The maximum length of transcripts was 15,689 bp, the minimum length was 201 bp, and the average length was 1079 bp. Among the 79,729 unigenes, 32,402 had a length of >500 bp and 47,327 had a length of <500 bp (Table 3).

#### 2.4.2. RNA-Seq Data Analysis and Unigene Function Annotation

**Figure 3:**
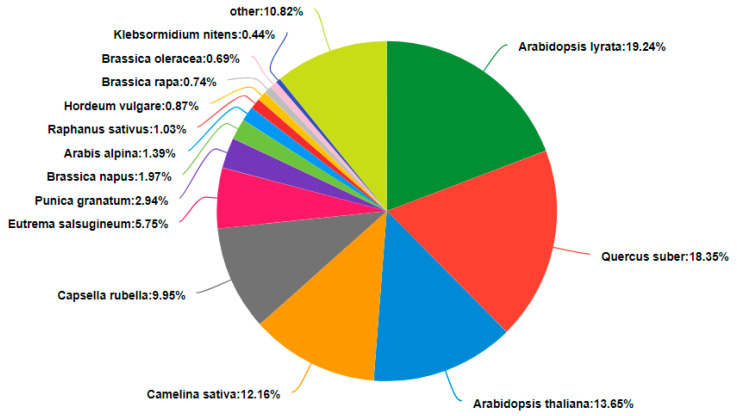
Species distribution of BLASTX matches for the R flixweed (*Descurainia sophia*) transcriptome unigenes.

**Figure 4:**
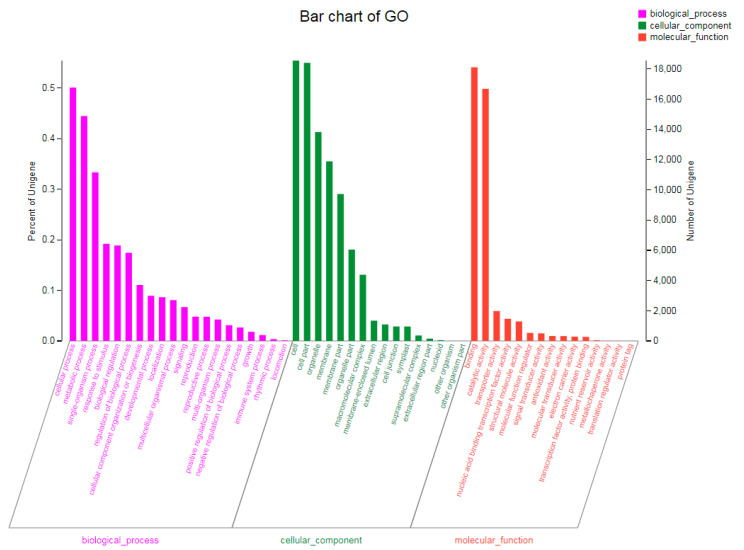
GO function classification of the annotated unigenes in R flixweed (*Descurainia Sophia*). The unigenes were allocated to three categories: biological process, cellular component, and molecular function.

**Figure 5:**
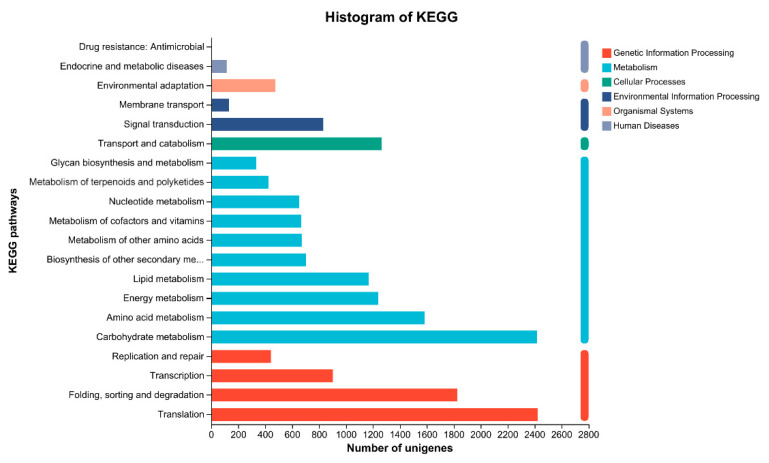
KEGG function classification of the annotated unigenes in the R population. The *y*-axis lists the various KEGG pathways; the *x*-axis indicates the number of unigenes.

Of the 79,729 high-quality unigenes, 54,117 were annotated in the six public databases. The highest number of annotated unigenes was found in the NR database, and the lowest number was found in COG (Table 4). According to the unigene annotations in the NR database, the unigene sequences of *D. sophia* were homologous with more than 14 species, with the greatest homology exhibited with *Arabidopsis lyrata* (19.24%) (Figure 3). A total of 33,476 unigenes were annotated in the GO database; these were classified into three categories as follows: biological process (BP), consisting of 20 subgroups; cellular component (CC), consisting of 16 subgroups; and molecular function (MF), consisting of 15 subgroups (Figure 4). In the BP category, cellular process (16,769) and metabolic process (14,884) were the most prevalent terms. In the CC category, cell (18,556) and cell part (18,406) were the most prevalent terms. In the MF category, binding (18,112) and catalytic activity (16,689) were the most prevalent terms. In addition, the 26,117 unigenes annotated in the KEGG database were classified into six first-category KEGG pathways and twenty second-category KEGG pathways; these pathways were mainly involved in the processes of translation, carbohydrate metabolism, folding, sorting, and degradation (Figure 5). 

#### 2.4.3. Candidate Dormancy Genes Selected in R Population

**Figure 6:**
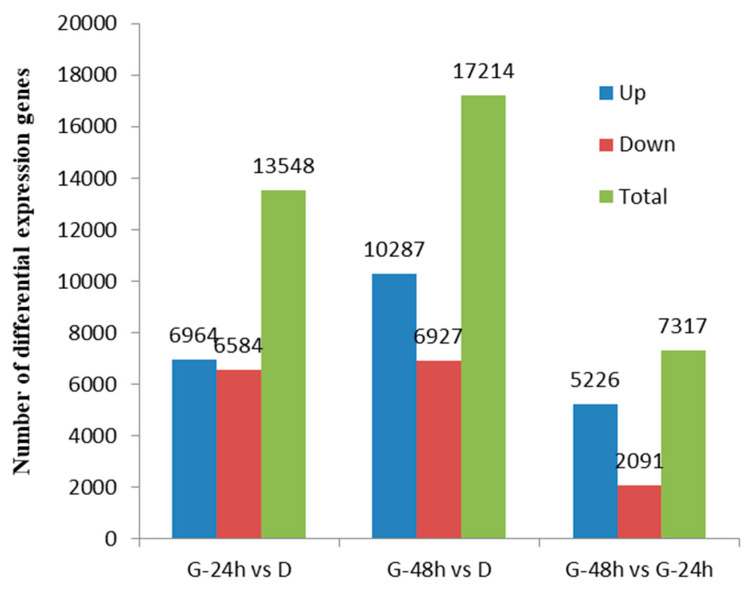
Number of differentially expressed unigenes in the D, G-24 h, and G-48 h treatments in comparison with the R population. D was a dormant-seed treatment; G_24 h and G_48 h were germinated-seed treatments.

**Figure 7:**
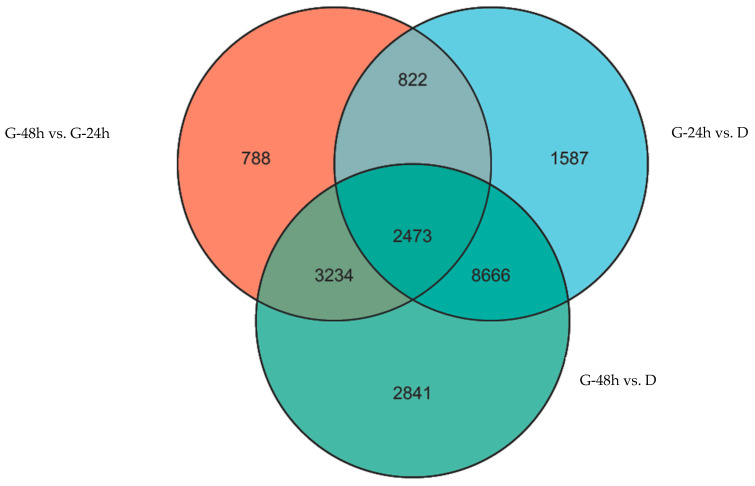
Venn diagram showing numbers of differentially expressed unigenes in the D, G-24 h, and G-48 h treatments in comparison with the R population.

The numbers of DEGs varied among the G-24 h vs. D, G-48 h vs. D, and G-48 h vs. G-24 h groups. There were 6964 upregulated DEGs and 6584 downregulated DEGs in the G-24 h vs. D group; 10,287 upregulated DEGs and 6927 downregulated DEGs in the G-48 h vs. D group; and 5226 upregulated DEGs and 2091 downregulated DEGs in the G-48 h vs. G-24 h group (Figure 6). A total of 2473 DEGs were present in all three groups, as illustrated in the Venn diagram in Figure 7. The results of GO functional-enrichment analysis showed that these 2473 DEGs could be enriched into 780 GO terms, classified as follows: 51 functional subgroups; 23 subgroups belonging to BP; 14 subgroups belonging to CC; and 14 subgroups belonging to MF. The 2473 DEGs were mainly enriched into 110 KEGG pathways, and the number of DEGs participating in the “plant hormone signal transduction” (map04075) pathway reached 72, this being the highest number recorded for any of the 110 pathways. 

The genes involved in ABA synthesis and signal transduction have previously been associated with seed dormancy [29,30]. The *DOG1* gene has also been reported to regulate seed dormancy [31]. According to function annotations and expression levels, eight candidate dormancy genes were selected from the R population and their expression was found to decrease continuously during seed germination (Table 5). Four out of the eight candidate dormancy genes were functionally enriched into the ABA signaling pathway and the plant hormone signal transduction pathway, and one of these was annotated as *CYP707A2*. All the remaining four candidate dormancy genes had the *DOG1* domain, with the function of controlling seed dormancy; two of these were annotated as the transcription factors *TGA4* and *TGA2*, and one was annotated as the cystathionine beta-synthase gene (Table 5). 

#### 2.4.4. Candidate Dormancy Genes Validated by qRT-PCR

The expression levels of the eight candidate dormancy genes were further validated by qRT-PCR using the same samples as those used for RNA-Seq. The results showed that five of the eight candidate dormancy genes had the same expression profiles as in RNA-Seq (relative to FPKM) during the whole process of seed germination in the R population. However, the expression level of the remaining three candidate dormancy genes first decreased and then increased in the course of R seed germination (Table 6). These results indicated that the remaining three candidate dormancy genes probably have other functions in the regulation of growth in plants of the *D. sophia* R population.

## 3. Discussion

A population of *D. sophia* that had evolved resistance to tribenuron-methyl was first reported in China in 2005 [32]. Since then, resistant *D. sophia* populations have continued to be reported [17,18,25,33]. Today, resistant *D. sophia* populations are widely distributed in wheat fields in China. High levels of resistance to ALS-inhibiting herbicides have been widely reported. The results of three recent studies [18,19,20] showed ALS mutations at positions 197, 376, and 574, respectively. In the present study, we also found a population with mutation at the ALS 197 position that had evolved high resistance to tribenuron-methyl, a result which further confirmed that weeds with mutations in the ALS conserved region have high resistance to ALS-inhibiting herbicides.

Zhou and Luo [16] reported that *D. sophia* seeds exhibited a high degree of dormancy and that this typically continues for a period of six months from ripening to natural germination. In their work, the *D. sophia* population was probably a susceptible biotype because no resistant *D. sophia* had been reported at that time. In the present study, however, we found that resistant and susceptible *D. sophia* populations had different dormancy periods. After seeds were harvested and stored at room temperature for the same number of days, we found that the seed germination rate of the resistant population was higher than that of the susceptible population. This showed that the resistant population exhibited a shorter period of dormancy compared with the susceptible population. This is the first report to identify a difference between resistant and susceptible *D. sophia* populations with respect to seed dormancy.

Next, we used RNA-Seq technology to discover eight candidate dormancy genes in the R population. These were selected from 2473 DEGs common to the following three groups: G-24 h vs. D; G-48 h vs. D; and G-48 h vs. G-24 h. One unigene was annotated as the homologous gene of *CYP707A2* (Table 6). It has previously been reported that *CYP707A2* controls seed dormancy in *A. thaliana*, with transcript levels having been found to increase between the late-maturation and full-maturity stages in dry seeds [34]. In other studies, it was found that the gene encoded ABA 8’-hydroxylase to regulate the ABA level and played a distinct role during the process of seed germination in *A. thaliana* [35,36]. In the present study, we found that the level of *CYP707A2* changed from 39.9 to 4.13 during the process of seed germination in *D. sophia*. This trend was also indicated by qRT-PCR validation analyses, further confirming that *CYP707A2* is a dormancy gene that controls seed germination. 

Additionally, in the present study, three unigenes were annotated as the transcription factors TGA2 and TGA4 as well as cystathionine beta-synthase (Table 6). To the best of our knowledge, no previous papers have reported any involvement of the genes *TGA4* and *TGA2* in the regulation of seed dormancy; however, TGA4 has been reported to regulate plant defense against pathogens [37,38], and TGA2 has been reported to take part in the redox signaling network in *A. thaliana* [39]. Cystathionine beta-synthase has been related to some diseases in humans, on account of its role in regulating the dormancy survival regulon in bacteria [40]. However, the role of cystathionine beta-synthase in the regulation of seed dormancy in plants has not been reported until now. The three unigenes were selected as candidate dormancy genes because of their *DOG1* domain, which has been associated with seed dormancy. However, further studies are required to determine fully whether the three unigenes have any seed dormancy function.

Wang et al. reported that transcription factor *BsTGAL6* upregulated the expression of *BsCYP81Q32* to induce non-target resistance to ALS-inhibiting herbicides in *Beckmannia syzigachne* [41]. It may be that *TGA4* and *TGA2*, the homologous genes of *BsTGAL6*, have the same function as *BsTGAL6* in regulating non-target resistance to tribenuron-methyl in *D. sophia*. It may also be the case that resistance to ALS-inhibiting herbicides in *D. sophia* is related to seed dormancy. Again, further studies are required to investigate the genes involved in the regulation of both resistance and dormancy.

In the present study, four unigenes could not be annotated as any known gene in the NR, Swiss-Prot, Pfam, COG, GO, or KEGG database. Any seed dormancy function in these unigenes needs to be identified using transgenic and key-gene-knockout technologies in a model plant species. From the above-mentioned analysis, we can see that the regulation mechanisms involved in seed dormancy in resistant *D. sophia* are very complicated. More research needs to be carried out to elucidate the regulation pathways associated with seed dormancy.

In summary, we found that a resistant *D. sophia* population (R) with the Pro-197-Thr mutation of ALS had evolved high resistance to tribenuron-methyl. Though seeds in resistant and susceptible (S) populations exhibited similar levels of vitality, the germination rate of the R population was higher than that of the S population, demonstrating that the seed dormancy period of the R population was shorter than that of the S population. RNA-Seq and qRT-PCR technologies were then applied in combination to select eight candidate dormancy genes from the R population. Among these, the expression level of *CYP707A2* continued to decrease during the seed germination process. Two unigenes were annotated as *TGA4* and *TGA2*, one unigene was annotated as the cystathionine beta-synthase gene, and four unigenes could not be annotated as any known gene in the six public databases. All eight candidate genes need to be further studied to determine whether they regulate seed dormancy pathways in the R populations, and the means employed if they do. This study is the first to elucidate the preliminary mechanisms involved in seed dormancy in a herbicide-resistant *D. sophia* population. The results of this study have theoretical significance for the development of control technologies for herbicide-resistant weeds.

## 4. Materials and Methods

### 4.1. Whole-Plant Bioassay of Suspected Resistant D. sophia Population

Seeds from the suspected resistant *D. sophia* population (R) were collected in 2016 from a winter wheat field in Shijiazhuang, Hebei Province, China, where tribenuron-methyl (benzoicacid,2-[(4-methoxy-6-methyl-1,3,5-triazin-2-yl)methylamino]carbonyl) had been applied annually and continually for more than 20 years. The *D. sophia* plants were grown normally at the field recommended dose of 22.5 g a.i. ha^−1^. The susceptible *D. sophia* population (S) was collected in 2016 from wasteland at Handan, Hebei Province, China, where winter wheat had never been planted and tribenuron-methyl had never been sprayed. 

Gibberellin (Jiangsu Fengyuang Biochemical Ltd., Sheyang, China) at 0.1% concentration was used to soak seeds of the R and S populations for 24 h to break their dormancy. Next, the seeds were washed with distilled water and planted in plastic pots of 15 cm diameter. The pots were placed into a greenhouse under night and day conditions of 15–18 °C and 20–25 °C, respectively, with natural lighting. Ten plants were retained in each pot when growth reached the 3–4-leaf stage.

Tribenuron-methyl was sprayed at doses of 0.1125, 1.125, 11.25, 112.5, and 1125 g a.i. ha^−1^ in the case of the R population, and 0.01125, 0.1125, 1.125, 11.25, and 112.5 g a.i. ha^−1^ in the case of the S population, using a moving-nozzle cabinet sprayer with a Teejet XR8003 flat fan nozzle. The spray volume was 400 L ha^−1^ at 275 kPa. In addition, there was a blank control treatment; in this case, water only was sprayed. After tribenuron-methyl had been sprayed for 21 days, the seedlings above ground were collected and measurements of fresh weight were taken. The experimental design involved the use of a completely randomized method with three biological replications; this was repeated two times.

The GR_50_ value was calculated according to the following equation:

$$y=C+\frac{D-C}{1+\exp\{b[\log(x)-\log({GR}_{50})]\}}$$

where *y* is the fresh weight of the plant as a percentage of the untreated control at dose x of tribenuron-methyl; *b* is the slope at the GR_50_; C and D are the minimum and maximum fresh weights of seedlings as a percentage relative to the untreated control; and GR_50_ is the dose of tribenuron-methyl that causes 50% inhibition in the fresh weight of plants (without roots). Statistical analysis was carried out using SAS/ATAT NLIN (Version 8.0).

### 4.2. Analysis of ALS Gene Sequence in R and S Populations

The mutations of ALS in the R population were detected in order to ensure that it was a highly resistant biotype. About 0.1 g of fresh leaf was taken from single plants in the S and R populations; these samples were then ground to powder in liquid nitrogen. DNA was extracted according to the instructions of the DNA extraction kit (BeiJing Cowin Biotech Co., Ltd., Beijing, China). Primers (forward: 5′-CGCTCCTCTCCTGAAGCTCACCA-3′; reverse: 5′-AAACAAACAGCAGTAGCGTCTGAAG-3′) were designed to amplify the whole coding region of the *ALS* gene. The PCR mixture contained 1 μL DNA template (50 ng), 0.5 μL of each primer (10 pmol μL^−1^), 12 μL 2 × Es Taq MasterMix (BeiJing Cowin Biotech Co., Ltd., Beijing, China), and 11 μL ddH_2_O so that a total volume of 25 μL was obtained. The PCR procedure was as follows: denaturation at 94 °C for 3 min; 33 cycles of 1 min at 94 °C, 1 min at 58 °C, and 1.5 min at 72 °C; 10 min at 72 °C; and, finally, holding at 4 °C forever. The *ALS* gene PCR amplification experiment was conducted using a BioRad engine (Hercules, CA, USA). 

The PCR products were linked to the pEASY-T3 cloning vector (BeiJing TransGen Biotech Co., Ltd., Beijing, China) and transformed into Trans1−T1 Phage-Resistant Chemically Competent Cell (BeiJing TransGen Biotech Co., Ltd.) to clone the *ALS* gene according to the instructions. The *ALS*-transgenic plasmids were sequenced at Beijing Ruibo Biotech Co., Ltd., using an ABI Prism 3730XL DNA sequencer. The *ALS* gene was cloned from three plants of each population. Finally, the mutation of ALS was analyzed using the DNAMAN software package (Version 6.0.3.48, Lynnon Biosoft, Vandreuil, QC, Canada) by alignment with the ALS sequence of *Arabidopsis thaliana* (NP_190425).

### 4.3. Differences in Seed Germination between R and S Populations

On 8 November 2017, seeds of the R and S populations were planted in plastic pots of 35 cm diameter, with 1 plant only in each pot. All pots were placed into a greenhouse under the conditions described above. The pots were watered every 3 days until the seeds ripened. On 12 June 2018, ripened seeds of the R and S populations were collected. The seeds of the R and S populations were immersed in water and 0.1%GA3, respectively, for 24 h, to break seed dormancy. All seeds were then washed 3 times with distilled water. Next, 50 seeds were placed into Petri dishes of 9 cm diameter with 1 layer of filter paper and 5 mL of distilled water. Dishes were then sealed with sealing film and placed into an artificial illumination incubator under conditions of 22 °C, a 16 h/8 h night/day cycle, and light intensity of 20,000 lx. After 7 days, the numbers of germinated seeds of the R and S populations were counted so that the respective seed germination rates could be determined; this ensured that the R and S seeds were still in the dormancy stage and exhibited the same good vitality.

Freshly ripened seeds of the R and S populations were then air-dried and stored in paper bags at room temperature (25 ± 5 °C). When the seeds began to germinate naturally on 25 December 2018, the seed germination rates of R and S populations were determined using the method described above. The experiments were carried out using a completely randomized design, with four biological replications.

### 4.4. Mining Candidate Dormant Genes from R Population Using RNA-Seq 

#### 4.4.1. RNA Extraction, cDNA Preparation, and Illumina Sequencing

Seeds of the R population were reproduced using the method described in Section 2.3 above. The freshly harvested dry seeds had strong dormancy; these formed the first treatment (D). Next, R seeds were treated with 0.1%GA3 for 24 h to break seed dormancy. The seeds were then washed 3 times with distilled water and placed into Petri dishes of 15 cm diameter with 1 layer of filter paper and 10 mL of distilled water. The Petri dishes were cultivated under the conditions described in Section 2.3 above. Embryos of R germinating seeds that began to break through after 24 h were collected as the second treatment (G-24 h). R seeds that germinated after 48 h were collected as the third treatment (G-48 h). Each treatment had 3 replications, and 9 samples were collected in total. The weight of each sample was about 2 g. All 9 samples were frozen with liquid nitrogen in a mortar and pestle to powder for the purpose of total RNA extraction. 

RNA purification, library construction, and sequencing were performed at Shanghai Majorbio Bio-pharm Biotechnology Co., Ltd. (Shanghai, China) according to the manufacturer’s instructions (Illumina, San Diego, CA, USA). Total RNA was extracted from the 9 samples using Plant RNA Purification Reagent according to the manufacturer’s instructions (Invitrogen, Carlsbard, CA, USA), and genomic DNA was removed using DNase I (TaKara). Next, the 2100 Bioanalyser (Agilent Technologies, Inc., Santa Clara, CA, USA) and ND-2000 (NanoDrop Thermo Scientific, Wilmington, DE, USA) were used to determine the integrity, purity, and quantity, respectively, of the total RNA. Finally, high-quality RNA samples (OD260/280 = 1.8~2.2, OD260/230 ≥ 2.0, RIN ≥ 8.0, 28 S:18 S ≥ 1.0, >2 μg) were prepared to construct a sequencing library. 

The RNA-seq transcriptome libraries of the 9 samples were prepared using the Illumina TruSeqTM RNA Sample Preparation Kit (San Diego, CA, USA). Double-stranded cDNA from each sample was synthesized using the SuperScript double-stranded cDNA synthesis kit (Invitrogen, Carlsbad, CA, USA) with random hexamer primers (Illumina), which used fragmented RNA as templates. The cDNA was then subjected to end-repair, phosphorylation, and ‘A’ base addition according to the Illumina library construction protocol. The 9 libraries were size-selected for cDNA target fragments of 200–300 bp on 2% Low Range Ultra Agarose; this was followed by PCR amplification. After quantification by TBS380, the 9 RNA-seq libraries were sequenced using the Illumina Hiseq xten sequencer (Illumina, San Diego, CA, USA).

#### 4.4.2. RNA-Seq Data Analysis and Genes Function Annotation

Raw paired-end reads from each library were trimmed and quality-controlled using SeqPrep (https://github.com/jstjohn/SeqPrep (accessed on 12 December 2018)) and Sickle (https://github.com/najoshi/sickle (accessed on 12 December 2018) with default parameters. Clean reads were then used to carry out de novo assembly using Trinity (http://trinityrnaseq.sourceforge.net/ (accessed on 12 December 2018)) [42]. All the assembled transcripts were searched for in 6 databases (NCBI protein nonredundant (NR), Swiss-Prot, Pfam, COG, GO, and KEGG) to identify those genes that had the highest sequence similarity with the given transcripts so that their function annotations could be retrieved [43,44]. 

#### 4.4.3. Candidate Dormancy Genes Selected in R Population

To identify differentially expressed genes (DEGs) between two compared treatments, the expression level of each transcript was calculated according to the fragments-per-kilobase of exon-per-million mapped reads (FRKM) method. RSEM (http://deweylab.biostat.wisc.edu/rsem/ (accessed on 12 December 2018)) was used to quantify gene and isoform abundance [45]. DESeq2 software 1.24.0 was utilized for differential expression analysis under conditions of q-value < 0.05 and |log_2_(Fold change)| ≥ 2. In addition, DEGs were subjected to GO and KEGG functional-enrichment analysis using Goatools (https://github.com/tanghaibao/Goatools (accessed on 12 December 2018) and Majorbio Cloud 2024, respectively [46]. 

Candidate dormancy genes were selected from DEGs, according to (1) their functional annotations associated with seed dormancy (*ABA* and *DOG1*) in NR, Swiss-Prot, Pfam, COG, GO, and KEGG databases; and (2) their expression levels continuously decreasing from the D treatment to the G-48 h treatment.

#### 4.4.4. Candidate Dormancy Genes Validated by qRT-PCR

Candidate dormancy genes in the R population were selected according to their statistical significance, their expression differences, and their annotations related to plant seed dormancy. The expression of each selected gene was detected using cDNA synthesized from RNA samples, which were used in the RNA-Seq experiment. *18SrRNA* was selected as the reference gene in *D. sophia* [47], and qRT-PCR primers of each candidate gene were designed according to the selected unigenes’ sequences using Primer Premier 5.0. The primer sequences are listed in Table 7.

The qRT-PCR was conducted on an iCycler iQ5 Real-Time PCR Detection System (Bio-Rad, Hercules, CA, USA) using the UltraSYBR Mixture (CWBIO, Beijing, China) following the manufacturer’s instructions. The reaction system contained 10 μL 2×UltraSYBR Mixture, 1 μL cDNA sample (50 ng), 0.5 μL of both forward and reverse primers (10 pmol μL^−1^), and 8 μL RNase-free ddH_2_O. Each cDNA sample had 3 biological replications. The qRT-PCR reaction program was as follows: 95 °C incubation 10 min, followed by 40 cycles of 95 °C for 15 s, 58 °C for 30 s and 72 °C for 30 s; melting curves were then performed from 55 °C to 95 °C with stepwise increases of 0.5 °C every 10 s. The relative expression of each candidate gene was calculated using the 2^−ΔΔCt^ method [48]. The qRT-PCR data variance analysis was conducted using Duncan’s new repolarization method in SPSS12.0.

## Figures and Tables

**Table 1:** Resistance levels in S and R populations.

Population	Herbicide	GR_50_ (g a.i. ha^−1^)	R/S
S	Tribenuron-methyl	0.12 ± 0.01	-
R		54.88 ± 5.25	457.34

Note: In this table, and those below, R—resistant population; S—susceptible population.GR_50_ was the herbicide dose that induced a 50% reduction in fresh weight in plants of flixweed (*Descurainia sophia*) populations. GR_50_ values are expressed as mean ± standard error. The R/S index was calculated as follows: GR_50_(R)/GR_50_(S).

**Table 2:** Differences in seed germination between R and S populations.

Population	Freshly Ripened Seeds Immersed in Water ^1^		Freshly Ripened Seeds Immersed in 0.1%GA3 ^2^		Seeds Allowed to Begin Germination Naturally ^3^	
	Number of Germinated Seeds	Seed Germination Rate (%)	Number of Germinated Seeds	Seed Germination Rate (%)	Number of Germinated Seeds	Seed Germination Rate (%)
S	0 ± 0 a	0	43 ± 2 a	86	3 ± 1 b	6
R	0 ± 0 a	0	44 ± 2 a	88	31 ± 3 a	62

The number of seeds used in each 9 cm diameter Petri dish was 50, with four biological replications. ^1^ The seeds were first immersed in water for 24 h and then placed into a Petri dish with a piece of filter paper and 5 mL distilled water. ^2^ The seeds were first immersed in 0.1%GA3 for 24 h and then placed into a Petri dish, as per the above-mentioned method, after washing with water. ^3^ The seeds beginning to germinate after naturally breaking dormancy were directly placed into a Petri dish, as per the above-mentioned method. Numbers of germinated seeds in both R and S populations were counted 7 days after being placed into Petri dishes. The germination value for each seed is expressed in the form of mean ± standard error. Where columns have the same letters, this means that there was no significant difference in the number of germinated seeds under the same conditions (*p* < 0.05).

**Table 3:** Assessments of de novo assembly of flixweed seeds using RNA-seq.

Types	Resource
Total raw reads	508,375,818
Total clean reads	504,976,046
Lowest number of clean reads	49,610,034
Highest number of clean reads	65,670,676
Total number of transcripts	164,087
Total number of unigenes	79,729
Number of unigenes > 500 bp	32,402
Number of unigenes < 500 bp	47,327
The longest length of transcript	15,689 bp
The shortest length of transcript	201 bp
Average length of transcripts	1079 bp
^1^ N50 of total transcripts	1665 bp
^2^ E90N50 of total transcripts	1796 bp
^3^ GC percent of total transcripts	42.30%

^1^ N50, 50% of the assembled bases were incorporated into transcript sequences with a length of N50. ^2^ For the expression in the top 90% of transcripts, 50% of the assembled bases were incorporated into transcript sequences with a length of N50. ^3^ The total number of G and C bases is expressed as a percentage of the total number of bases.

**Table 4:** Sequence annotations of the R flixweed (*Descurainia sophia*) transcriptome.

Public Database	Unigene Number	Percentage
Annotated in NR	47,188	59.19
Annotated in Swiss-Prot	42,229	52.97
Annotated in Pfam	35,841	44.95
Annotated in COG	16,174	20.29
Annotated in GO	33,476	41.99
Annotated in KEGG	26,117	32.76
Annotated in at least one of above-mentioned databases	54,117	67.88
Annotated in none of the above-mentioned databases	25,612	32.12
Total	79,729	-

**Table 5:** Genes potentially associated with seed dormancy in R flixweed (*Descurainia sophia*) via RNA-Seq.

Gene_id	Function Annotations	E-Value	FPKM		
			Treatments		
			D	G_24 h	G_48 h
TRINITY_DN26001_c0_g1	Response to abscisic acid and plant hormone signal transduction	6.00 × 10^−71^	381.35 ± 1.39 a	34.00 ± 12.26 b	4.34 ± 0.76 c
TRINITY_DN27555_c0_g1	Abscisic acid-activated signaling pathway and plant hormone signal transduction	2.60 × 10^−64^	82.97 ± 4.40 a	2.61 ± 2.03 b	0.27 ± 0.14 b
TRINITY_DN30509_c3_g1	Abscisic acid-activated signaling pathway and plant hormone signal transduction	1.10 × 10^−127^	938.69 ± 32.82 a	105.23 ± 34.59 b	47.70 ± 4.83 b
TRINITY_DN32745_c2_g1	Abscisic acid 8′-hydroxylase 2 and *CYP707A2*	4.00 × 10^−55^	39.90 ± 3.43 a	16.89 ± 5.19 b	4.13 ± 0.23 c
TRINITY_DN25776_c0_g1	Transcription and seed dormancy control	2.60 × 10^−101^	155.41 ± 2.18 a	5.34 ± 1.95 b	1.08 ± 0.12 b
TRINITY_DN25783_c0_g1	Transcription factor TGA4 and seed dormancy control	8.00 × 10^−68^	40.97 ± 0.30 a	10.09 ± 3.24 b	1.93 ± 0.53 c
TRINITY_DN28379_c0_g1	Transcription factor TGA2.3-like isoform X1 and seed dormancy control	8.70 × 10^−60^	55.19 ± 0.93 a	13.32 ± 4.58 b	3.82 ± 0.43 b
TRINITY_DN36452_c0_g1	Cystathionine beta-synthase and seed dormancy control	3.30 × 10^−173^	1011.72 ± 29.45 a	80.95 ± 25.94 b	36.95 ± 5.02 b

Identification of candidate dormancy genes in R population via RNA-Seq, *p*-value < 0.05 and log_2_(Fold change) ≥ 2. E-Value indicates the probability of a chance search, i.e., the lower the value, the more credible the result. FPKM—fragments-per-kilobase of transcript sequence per million base pairs sequenced. Each FPKM value is expressed as the mean ± standard error. D was a dormant-seed treatment; G_24 h and G_48 h were germinated-seed treatments. Where rows have the same letters, this indicates no significant difference in FPKM (*p* < 0.05).

**Table 6:** The relative expression of genes potentially related to seed dormancy in R *Descurainia sophia*, obtained using the qRT-PCR(2^−ΔΔCt^) method.

Gene_id	Function Annotations	qRT-PCR (2^−ΔΔCt^)		
		Treatments		
		D	G_24 h	G_48 h
TRINITY_DN26001_c0_g1	Response to abscisic acid and plant hormone signal transduction	1 a	0.0449 ± 0.0100 b	0.0102 ± 0.0011 c
TRINITY_DN27555_c0_g1	Abscisic acid-activated signaling pathway and plant hormone signal transduction	1 a	0.0057 ± 0.0008 b	0.0086 ± 0.0024 b
TRINITY_DN30509_c3_g1	Abscisic acid-activated signaling pathway and plant hormone signal transduction	1 a	0.0285 ± 0.0047 b	0.0281 ± 0.0065 b
TRINITY_DN32745_c2_g1	Abscisic acid 8′-hydroxylase 2 and *CYP707A2*	1 a	0.1373 ± 0.0078 b	0.0802 ± 0.0140 c
TRINITY_DN25776_c0_g1	Transcription and seed dormancy control	1 a	0.0079 ± 0.0059 b	0.0072 ± 0.0011 b
TRINITY_DN25783_c0_g1	Transcription factor TGA4 and seed dormancy control	1 a	0.0412 ± 0.0131 b	0.0462 ± 0.0136 b
TRINITY_DN28379_c0_g1	Transcription factor TGA2.3-like isoform X1 and seed dormancy control	1 a	0.0486 ± 0.0139 b	0.0257 ± 0.0118 b
TRINITY_DN36452_c0_g1	Cystathionine beta-synthase and seed dormancy control	1 a	0.0061 ± 0.0013 b	0.0145 ± 0.0079 b

When the qRT-PCR(2^−ΔΔCt^) method was used, the expression of potential genes in the D treatment was regarded as 1 in all cases. Where rows have the same letters, this indicates no significant difference in the relative expression of the potential genes (*p* < 0.05).

**Table 7:** The primers used in qRT-PCR.

Gene_id	Forward Sequence (5′ to 3′)	Reverse Sequence (5′ to 3′)
TRINITY_DN26001_c0_g1	AAAGGAGGAAGATGAAGGAA	CTCGGATCACAGTTACAAAGC
TRINITY_DN27555_c0_g1	GGACTTCCTGCGGGATTTAG	CTCCACCACCACCGTCTTCT
TRINITY_DN30509_c3_g1	CACCTACACCCATAGCCTGAC	ACATCCCACTAACCCTAAATC
TRINITY_DN32745_c2_g1	CGAGGGTGTTGATGGACTTT	TCCTTCACGCCTTCTGCTCT
TRINITY_DN25776_c0_g1	TGCTATGGATGGGAGGAT	GTCGCGGTAAGATCACTC
TRINITY_DN25783_c0_g1	CTTCCTCCGTAACAACAA	CAATCTCCCAAGTCCTAA
TRINITY_DN28379_c0_g1	TAAGAAACTCCGCTTGTTGG	TAAGAAACTCCGCTTGTTGG
TRINITY_DN36452_c0_g1	GATGGCGTCTTGAATCCC	AAGGCACAACGACTTAGGTAT
*18SrRNA* (reference gene)	TAGTTGGTGGAGCGATTTGTCTG	CTAAGCGGCATAGTCCCTCTAA

## Data Availability

Data are contained within the article.
